# Assessing the Relationship between Sources of Stress and Symptom Changes among Persons with IBD over Time: A Prospective Study

**DOI:** 10.1155/2016/1681507

**Published:** 2016-10-04

**Authors:** Matthew T. Bernstein, Laura E. Targownik, Kathryn A. Sexton, Lesley A. Graff, Norine Miller, John R. Walker

**Affiliations:** ^1^Department of Psychology, University of Manitoba, Winnipeg, MB, Canada; ^2^IBD Clinical and Research Centre, Winnipeg, MB, Canada; ^3^Department of Internal Medicine, University of Manitoba, Winnipeg, MB, Canada; ^4^Department of Clinical Health Psychology, University of Manitoba, Winnipeg, MB, Canada

## Abstract

*Objective*. To describe the sources of stress for persons with IBD and changes with changes in symptoms.* Methods*. 487 participants were recruited from a population-based IBD registry. Stress was measured at study entry and three months later, using a general stress measure and the Sources of Stress Scale. Four symptom pattern groups were identified: persistently inactive, persistently active, inactive to active, and active to inactive.* Results*. General stress levels were stable within each symptom pattern group over the three-month period, even for those with changing symptom activity. The persistently active group had higher general stress at month 0 and month 3 than the persistently inactive group and higher mean ratings of most sources of stress. IBD was rated as a highly frequent source of stress by 20–30% of the persistently active group compared to 1-2% of the inactive group. Finances, work, and family were rated as high frequency stresses in the persistently active group at a similar level to IBD stress. In the groups with fluctuating symptoms, there was little change in stress ratings with changes in symptom activity.* Conclusion*. Stress was experienced across several domains in addition to stress related to IBD. Persons with active symptoms may benefit from targeted stress interventions.

## 1. Introduction

Inflammatory bowel diseases (IBD), generally classified as Crohn's disease and ulcerative colitis, are chronic inflammatory diseases that affect the gastrointestinal tract. Stress can impact many aspects of human physiology, including the release of proinflammatory mediators, sympathetic nerve output, and intestinal permeability, all of which are involved in the pathophysiology of IBD [1]. However, the relationship between a person's stress experience and their disease activity and symptoms is complex [2]. Perceived stress is related to many characteristics of the individual. In a nationally representative US sample, perceived stress was found to be higher among women, younger adults, the unemployed, and those with lower levels of education and income [3].

The available research exploring the role of stress in IBD suggests that stress may be a factor in the exacerbation of IBD-related symptoms [2, 4, 5]; the relationship between stress-related IBD symptoms and intestinal inflammation is less well understood. Conversely, having ongoing IBD-related symptoms is a potential psychological stressor, and therefore an increase in stress may be a consequence of IBD exacerbation. The extent of change in stress with change in symptom activity has not been well described in previous research, much of which is cross-sectional [5].

We have previously demonstrated that persons with ongoing IBD-related symptom activity were more likely to report higher levels of stressful experiences than those with stable inactive symptoms, identifying work, financial, and family stress as the most common sources of stress [6]. When participants experienced a symptom exacerbation they were more likely to describe their IBD as a life stress. Interestingly, participants with and without active symptoms reported a wide range of stressors. However, this was an exploratory study using open-ended questions so it was not possible to systematically examine sources of stress and the magnitude of perceived stress in each area.

In the current study, we aimed to describe and quantify the sources of stress experienced by persons with IBD relative to their IBD symptom activity, exploring changes in the types and amount of stress occurring concomitantly with changes in symptom level. This study builds on the previous work by Singh and colleagues [6] by having participants rate the frequency of stress in each of the ten most common stress areas identified in that prior study. It was expected that this approach would provide a more complete understanding of the sources of stress experienced by persons with IBD, with the potential to guide stress management strategies.

## 2. Methods

### 2.1. Participants

This research was part of a larger prospective cohort study of the relationships among perceived stress, symptoms, and inflammation (assessed by fecal calprotectin) in persons with IBD [7]. Participants were recruited from the University of Manitoba IBD Research Registry, a validated population-based database [8]. The registry enrolled individuals with IBD identified through the administrative health database of Manitoba Health, the single insurer that provides comprehensive health care to all residents of Manitoba. The registry includes approximately half of all Manitobans with IBD and has been previously shown to be similar in demographic characteristics to the overall population with IBD [9]. All those in the registry who were over the age of 18 and had current addresses within Manitoba (*N* = 1958) were invited by letter to participate with contacts starting in January 2012. The letter explained that the survey would include questions about personal characteristics and information regarding their IBD and would also require the collection of stool samples on three occasions that were to be mailed in special containers to the research centre. Those who chose to participate were sent survey materials on two separate occasions 3 months apart. This study was approved by the University of Manitoba Research Ethics Board.

### 2.2. Variables/Measurement

#### 2.2.1. Symptom Activity

The Manitoba Inflammatory Bowel Disease Index (MIBDI) [10] is a brief, validated index developed for cohort studies to identify the participant's symptom activity over a 6-month period. The MIBDI has been adapted for use in a 3-month time period [7]. It uses a 6-level response format and symptom frequency anchors to ensure more precise reporting. Active symptoms were defined as reporting one of 3 levels of IBD symptoms, from constantly (daily) to sometimes (a few days every other week), for the previous three months. Inactive symptoms were defined as reporting one of the other three levels, that is, symptoms occasionally (1-2 days each month) to not at all, in the previous three months. The MIBDI has a high degree of sensitivity and specificity in identifying persons with active disease according to disease specific symptom measures—the Harvey-Bradshaw for Crohn's disease and the Powell-Tuck for ulcerative colitis [10]. It also has high specificity and sensitivity for identifying persons identified as having active disease on the Inflammatory Bowel Disease Questionnaire—a quality of life measure [10].

#### 2.2.2. Stress

We assessed both the types of stressors and level of general stress. Regarding the types of stressors, we used the Sources of Stress Scale, which was developed for this study by categorizing the range of stressors identified by IBD participants in the Singh et al. study [6]. The scale lists 10 potential types of stressors including (1) your IBD, (2) other health concerns (non-IBD), (3) financial concerns, (4) work or school concerns, (5) family situations, (6) a separation or break-up in a relationship, (7) conflict with someone close, (8) important life events like a family wedding, job change, or new baby, (9) the death or possible death of someone close, and (10) other stressors (participants were asked to list these). For each stressor, participants were asked to rate how often they experienced that type of stress over the last month, using a 5-point Likert type scale, with anchors of 0 (*none of the time*), 1 (*a little of the time*), 2 (*some of the time*), 3 (*most of the time*), and 4 (*all of the time*). Many stressors in life are chronic (e.g., financial problems) rather than associated with a specific life event. Stressors that were rated most or all of the time (3 or 4) were considered to indicate high frequency stress about that theme.

We used Cohen's Perceived Stress Scale-14 (CPSS-14) [11] to assess the level of general stress. The CPSS is a 14-item instrument, which is designed to measure the degree to which day-to-day events and encounters are interpreted as being stressful or which overwhelm one's ability to cope. High CPSS scores have been shown to be associated with poor quality of life and poor coping in a variety of disease states [12, 13].

### 2.3. Statistical Methods

In order to facilitate comparisons between persons whose symptom activity changed from month 0 to month 3, compared to persons who indicated no change in symptom activity across time, participants were divided into four symptom activity groups, two of which were reflecting stable symptom patterns,* persistently inactive (inactive MIBDI at months 0 and 3) and persistently active (active MIBDI at month 0 and month 3)*, and two of which reflected fluctuating symptom patterns,* inactive to active (inactive MIBDI at month 0 and active MIBDI at month 3) and active to inactive (active MIBDI at month 0 and inactive MIBDI at month 3)*. We compared the mean general stress (CPSS) scores among the 4 symptom activity groups, at month 0 and month 3. We also compared the mean rating scores of each of the 10 stress categories at month 0 and month 3 within each of the symptom activity groups. All mean scores are presented with 95% confidence intervals in Tables 1 [Table tab2]–3. Confidence intervals are typically used in survey research and they allow for convenient comparisons within and across groups and time periods within tables and also across groups in Tables 1 [Table tab2]–3. Confidence intervals have been recommended rather than pairwise significance tests for comparisons between and within groups because they help the reader understand the magnitude of differences rather than simply concluding that a difference is statistically significant [14, 15]. When making comparisons between means (i.e., between groups and across different question items), it should be noted that in approximately 1 case out of 20, the 95% confidence intervals will be nonoverlapping even in the absence of a difference in that measure within the underlying populations. In considering missing data, respondents who missed more than 2/3 of the items on the CPSS (*N* = 4) were eliminated from the analysis. If a respondent missed some responses on the CPSS but completed more than 2/3 of the items (*N* = 9) their score was prorated to adjust for the missing responses and provide an accurate total score. The formula used in prorating for the 14-item scale was ((sum of items answered) *∗* 14)/number of items answered. Respondents who did not complete all of the items on the Sources of Stress Scale were excluded from the analysis of that scale (*N* = 7). The number of participants included in each analysis is indicated in each table.

## 3. Results

Overall, 487 participants completed the month 0 survey and 432 the month 3 survey. Only the 432 who completed both surveys were included in the analyses. 63% of the sample was female, and 54% had Crohn's disease, while 46% had ulcerative colitis. The mean age of this sample was 55.4 years (SD = 13.16), and the mean number of years since diagnosis was 22.0 years (SD = 10.98) for a mean age at diagnosis of 33.4 years. The clinical characteristics of the sample are described in more detail in a previous paper by our group [7].

Table 1 shows the mean general stress (CPSS) scores for individuals across the stable and fluctuating symptom activity groups. In evaluating stress level, we considered the persistently inactive symptom group as the reference group, because previous research [16] suggests that the stress level for this group is similar to persons without IBD. Those with persistently active symptoms had higher mean general stress levels at month 0 and month 3 than those with persistently inactive symptoms as indicated by the nonoverlapping 95% confidence intervals (means and confidence intervals that are not overlapping in the corresponding month are highlighted in bold). Similarly, in the fluctuating symptom groups, when participants were experiencing active symptoms (either at month 0 or month 3), the ratings of general stress were higher than for those of the persistently inactive group, as indicated by the nonoverlapping confidence intervals. We also considered whether there were changes in general stress from month 0 to month 3 within each of the symptom pattern groups by identifying when the confidence intervals were not overlapping. Overall, general stress levels were stable for each symptom pattern group over the three-month time period (as indicated by overlapping confidence intervals) with high correlations between month 0 and month 3 measurements within each group (*r* = .71–.78). This was true even for the fluctuating groups, where we might have expected a change in general stress when their symptoms changed from inactive to active or vice versa.

Table 2 compares the frequency ratings of the 10 sources of stress (using the Sources of Stress Scale) across the two time periods (month 0 and month 3) for the persistently inactive and persistently active groups. Comparing the persistently active group to the persistently inactive group, there were higher mean ratings of almost every source of stress (indicated in bold). IBD was rated as a high source of stress by 20–30% of respondents with active symptoms as compared to only 1-2% of those with inactive symptoms. Many of the other sources of stress were reported to be as high as stress related to IBD in the active symptom group including other health concerns, finance, work, and family—all described as highly frequent by 20 to 30% of respondents. We also considered whether there were changes in each source of stress from month 0 to month 3 within each of the symptom pattern groups by examining whether the confidence intervals were not overlapping. There was only one comparison where the confidence intervals for the mean ratings did not overlap, indicating a lower level for other stressors in month 3 than month 0 in the persistently active symptom group. Taking into account the large number of comparisons in this table (10 for each group), this may have been a chance finding given the 95% confidence intervals. Generally, the mean ratings of sources of stress were stable over the three-month time period.

Table 3 describes the frequency ratings of the 10 sources of stress across the two time periods (month 0 and month 3) for the fluctuating symptom groups—inactive to active and active to inactive. We compared these groups to the persistently inactive symptom group in Table 2; means and confidence intervals that do not overlap with those of the persistently inactive group are indicated in bold. Both fluctuating symptom groups reported higher stress related to IBD during the active symptom period than during either period for the inactive symptom group. Unexpectedly, higher stress related to IBD was also reported within the fluctuating symptom groups during the* inactive* symptom period, when compared with the group with persistently inactive symptoms. Those in the inactive to active group reported higher stress in the inactive period (month 0) compared to the persistently inactive group in the areas of finance, work, and family stresses. This group did not differ from the persistently inactive group at month 3 in frequency of stress in these areas when their symptoms were active. The active to inactive group differed from the persistently inactive group in stress about other health concerns and about family during both active and inactive time periods (months 0 and 3). They also differed in rating of conflict with someone close during the inactive time period (month 3).

We also considered whether there were significant changes in each source of stress from month 0 to month 3 within each of the fluctuating symptom pattern groups (i.e., nonoverlapping confidence intervals). We would have expected that with changes in symptom activity there would be changes in stress, but no changes in frequency of these sources of stress were identified. Looking at the inactive to active group, while IBD was rated as a highly frequent stressor by 20% of respondents, there were a number of other sources of stress that were rated by a similar proportion of respondents, including 23% who indicated finances as a highly frequent source of stress and 14% who indicated family. During the active symptom period of the active to inactive group, while 20% indicated IBD as a highly frequent source of stress, similar proportions rated other health concerns (25%), work (20%), family (25%), and conflict with someone close (19%) as high sources of stress.

## 4. Discussion

This study aimed to systematically explore the specific domains persons with IBD experience as their main sources of stress and to consider whether their perception of these stressors changes over time and in concert with changes in symptom activity. The stability of general stress over the three-month time period in this study, as indicated by the mean levels and correlations across the three-month time period, was striking within all four of the symptom pattern groups. Among those who changed symptom status from inactive to active or from active to inactive, there was no significant change in general stress. We found that general stress was higher in persons with persistently active compared to those with persistently inactive symptoms, which is consistent with previous research [16]. Comparing the mean levels to normative values, perceived stresses in persons with inactive symptoms were similar to those which have been reported in community samples of persons without IBD [16].

Persons with IBD reported stress related to a range of different domains. Not surprisingly, stress related to IBD concerns was low for persons experiencing stable, inactive symptoms and much higher for those reporting persistently active symptoms.

Ratings of stress about other common issues (work, family, and finances) were also considerably higher in those with active symptoms than among those with inactive symptoms. This finding is consistent with the influential cognitive-transactional model of stress developed by Lazarus and Folkman [17]. In this view, one's evaluation of stress is influenced by the environmental stressor, one's appraisal of their ability to manage the stress, and the coping strategies used or perceived to be available. It would thus not be surprising if the appraisal of one's ability to manage stresses was influenced by worsening of health problems. Managing life's challenges becomes more difficult when feeling unwell and troubled by symptoms. It is also consistent with a recent Canadian clinic and web-based survey of persons with IBD [18], which found that many persons reported a negative impact of IBD on leisure activities (64%), interpersonal relationships (52%), and financial security (40%).

IBD was only a highly frequent source of stress (experienced most or all of the time) in 20 to 30% of those whose IBD became active symptomatically. This suggests that most persons with IBD may develop a sense of mastery or at least familiarity with their disease such that even when it becomes symptomatic, the majority does not perceive it as frequently stressful. It is likely that most of those in this community sample were experiencing moderate levels of symptoms. Those who experience more severe symptoms are likely to experience considerably higher level of stress around symptoms than those who have moderate levels of symptoms.

Other than concern about IBD, the highest frequency sources of stress in all four symptom groups were concerns about health (not related to IBD), work, family, and financial stress. Most epidemiological research has focused on one or two sources of stress at a time (such as work stress or relationship stress), and there are few studies that have examined a fuller range of sources of stress. The studies that have considered a broader range of stressors report that work, family, and finances are common sources of stress and concern in the general community [19, 20].

For persons who shifted from inactive to active symptoms compared to those with persistently inactive symptoms, concern about IBD symptoms was higher at baseline. When this group reported having active symptoms three months later, there was minimal change in IBD symptom related stress. One might have expected persons reporting inactive IBD symptoms to be more stressed about their IBD, when their symptoms became active three months later. A similar pattern was noted for the other sources of stress, with higher ratings of other important areas (finance, work, and family) at month 0 in this group than in the persistently inactive group and then little change in frequency of stress when later experiencing active symptoms at month 3. There are a number of potential explanations of this phenomenon. First, those persons transitioning from inactive to active symptoms could have had recent experiences of problematic IBD symptoms, leading them to have more concern about their IBD even when they were not yet having active symptoms. We were unable to test this hypothesis with our data; however, it is conceivable that the fluctuating nature of these participants' symptoms is more characteristic of their IBD experience in general compared to the participants whose IBD symptoms were more stable over the period of the study. Second, these individuals may have been experiencing a higher level of symptoms at month 0 than those in the persistently inactive group, which was not captured in the inactive/active symptom categorization. We explored this possibility by comparing the scores on the 6-point MIBDI symptom scale at month 0 between the persistently inactive group and the inactive to active symptom group. We found that those in the inactive to active symptom group had a higher frequency of symptoms at month 0 than the persistently inactive group (data not reported here but available on request), providing some support to this explanation. On the other hand, even the highest symptom rating in the inactive group (the cut point on the MIBDI scale for the inactive group) was quite low—symptoms occasionally (1-2 days each month). Third, this inactive to active symptom pattern group also provided higher ratings of stress related to work, family, and finances in the period before switching to active symptoms. This suggests that persons in this group may have been experiencing higher stress overall before the change to active symptoms, although it is not clear why their concern about IBD would be higher in a period of relatively inactive symptoms.

Evaluating these and other possible explanations of these findings will require additional research on the relationship between stress and IBD symptoms. This suggests the importance of studying stress in periods before and after persons with IBD experience active symptoms. Considering persons who shifted from active to inactive symptoms, there was once again no change in IBD-related stress, nor were there any fluctuations in the ratings of stress related to work, family, and finances. We were not able to determine whether there would have been a further reduction in stress with continued relief from active symptoms over a longer time period.

This study has a number of limitations, which should be kept in mind in interpreting the results. The response rate to the invitation to participate sent to persons in the Manitoba IBD Registry was relatively low (24.9%). This study was part of a larger survey project [6] in which participants agreed to respond to three surveys over 6 months and supply three stool samples. The demands of the study likely reduced participation relative to less demanding survey studies with no requirements for stool samples. Most of the participants in our sample (and in the population) had been diagnosed with IBD many years earlier and consequently may have had the opportunity to develop effective coping strategies and treatment for IBD. Persons with a very recent diagnosis of IBD likely have different views of the stress related to having IBD symptoms and different experiences with stress in general.

Another limitation of the study is our reliance on a global symptom activity measure, the MIBDI. While the MIBDI correlates well with more detailed indices of IBD symptom severity such as the Harvey-Bradshaw and Powell-Tuck scores [10], the difference in level of stress associated with each level of symptom frequency on the MIBDI may not reflect even intervals. That is, a change from a score of three to four (three was our cutoff for active symptoms) may not have the same implications for stress burden as a larger change in symptom frequency, such as a change from 1 to 4. Further, some IBD symptoms may be more difficult to cope with than others and this is not well captured by more global IBD symptom measures. For instance, there are likely more dramatic changes in stress levels for patients who go from having very limited symptoms to having severe symptoms such as bloody diarrhea and pain or for patients who go from being severely symptomatic to being very much improved (e.g., with medication treatments) versus somewhat improved. It is difficult to capture these patterns of symptom change in a population-based study and may be easier to capture this in a clinic-based study, where participants are more likely to have more active symptoms. Finally, the small size of the fluctuating symptom pattern groups may have limited the power of statistical comparisons involving these groups. In spite of these limitations, this study's use of a population-based community sample and a prospective cohort design provides us with insights into the relationship between symptomatic activity and types of stress across the breadth of the IBD patient experience.

Overall, levels of both general stress and specific sources of stress remained fairly stable regardless of changes in symptomatic activity over time. While IBD is seen as a source of highly frequent stress more often in those with active or fluctuating symptoms, they may also be experiencing heightened stress in relation to work, family, and financial stress. Persons with active or fluctuating IBD may therefore benefit from a review of the sources of stress in their lives, and those with high stress are likely to benefit from more targeted stress management interventions. The stress measures used in this study were developed for research and it is not necessary to use them in everyday clinical practice to assess level of stress. A simple question during the clinical encounter such as “Have you been having any difficulties with stress related to IBD or other factors in your life?” may help identify concerns that should be addressed, including concerns about the disease and its treatment. Since stress correlated with symptoms in this study, the possibility that stress reduction may positively impact symptom experience should be further explored.

## Figures and Tables

**Table 1:** Level of general stress (CPSS score) at month 0 and month 3 for participants with stable (inactive or active) or fluctuating (increasing or decreasing) symptom patterns.

Symptom pattern	Month 0 mean general stress (95% CI)	Month 3 mean general stress (95% CI)	Correlation of general stress at months 0 and 3 (95% CI)
Persistently inactive (*n* = 262)	18.12 (17.10, 19.14)	17.46 (16.46, 18.45)	.71 (0.64, 0.76)
Inactive to active (*n* = 35)	20.84 (18.01, 23.67)	**21.71 **(18.50, 24.93)^a^	.78 (0.61, 0.88)
Active to inactive (*n* = 37)	**23.31 **(20.24, 26.37)^a^	**22.19 **(19.14, 25.24)^a^	.72 (0.52, 0.85)
Persistently active (*n* = 98)	**23.62 **(21.77, 25.47)^a^	**23.64 **(21.81, 25.46)^a^	.74 (0.61, 0.80)

^a^Means and confidence intervals that do not overlap with the corresponding mean rating and confidence intervals at that month in the persistently inactive symptom pattern group are indicated with this superscript and bold font.

**Table 2 tab2:** Sources of stress for those with persistently inactive symptoms and those with persistently active symptoms (month 0 and month 3).

Sources of stress	Persistently inactive symptom pattern (*n* = 262)				Persistently active symptom pattern (*n* = 93)			
	Mean rating frequency of stressor		Proportion reporting high frequency		Mean rating frequency of stressor		Proportion reporting high frequency	
	Month 0	Month 3	Month 0	Month 3	Month 0	Month 3	Month 0	Month 3
IBD	.64 (.55, .73)	.68 (.59, .79)	1.2%	2.3%	**1.98 **(1.77, 2.18)^a^	**1.85 **(1.65, 2.05)^a^	31.2%	21.4%
Other health	1.02 (.91, 1.14)	1.04 (.92, 1.16)	8.5%	8.0%	**1.44 **(1.21, 1.66)^a^	**1.47 **(1.24, 1.70)^a^	18.1%	20.4%
Finance	.97 (.85, 1.09)	.90 (.79, 1.02)	6.9%	5.7%	**1.68 **(1.43, 1.93)^a^	**1.55 **(1.29, 1.80)^a^	28.7%	28.9%
Work/school	.94 (.81, 1.08)	.94 (.81, 1.08)	10.4%	12.1%	**1.32 **(1.05, 1.59)^a^	**1.22 **(.97, 1.48)^a^	22.2%	16.3%
Family	1.22 (1.10, 1.34)	1.11 (.99, 1.24)	8.9%	10.3%	**1.95 **(1.73, 2.17)^a^	**1.79 **(1.53, 2.04)^a^	33.0%	30.6%
Relationship separation	.14 (.07, .20)	.14 (.09, .20)	1.2%	.40%	**.44 **(.22, .66)^a^	.37 (.19, .54)	7.6%	5.3%
Conflict with someone close	.71 (.60, .82)	.57 (.48, .67)	5.0%	1.9%	**1.20 **(.96, 1.45)^a^	**1.10 **(.85, 1.36)^a^	19.4%	14.4%
Important life events	.45 (.35, .55)	.56 (.45, .67)	3.1%	4.6%	.**78 **(.57, .99)^a^	**.74 **(.52, .97)^a^	5.6%	9.3%
Death or possible death of someone close	.57 (.45, .68)	.55 (.44, .66)	5.0%	4.2%	.81 (.58, 1.03)	**.98 **(.73, 1.24)^a^	8.6%	13.4%
Other stressors	.56 (.43, .69)	.53 (.41, .64)	8.2%	5.7%	**1.09 **(.81, 1.36)^a^	.53 (.31, .75)^b^	19.6%	10.2%

*Note*. Each source of stress was rated on a 5-point scale that included the following anchors: 0 (*none of the time*), 1 (*a little of the time*), 2 (*some of the time*), 3 (*most of the time*), and 4 (*all of the time*). Highly frequent stress was considered a rating of 3 or 4 (most or all of the time) on this rating scale.

^a^Means and confidence intervals that do not overlap with the corresponding mean rating and confidence intervals at that month in the persistently inactive symptom pattern group are indicated with this superscript and bold font.

^b^Month 0 and month 3 confidence intervals do not overlap within this group.

**Table 3:** Sources of stress for those with fluctuating symptoms from month 0 to month 3.

Sources of stress	Inactive to active symptom pattern (*n* = 35)				Active to inactive symptom pattern (*n* = 35)			
	Mean rating frequency of stressor		Proportion reporting high frequency		Mean rating frequency of stressor		Proportion reporting high frequency	
	Month 0	Month 3	Month 0	Month 3	Month 0	Month 3	Month 0	Month 3
IBD	**1.17 **(.89, 1.45)^a^	**1.66 **(1.31, 2.00)^a^	5.7%	20.0%	**1.77 **(1.37, 2.17)^a^	**1.38 **(1.05, 1.71)^a^	20.0%	13.5%
Other health	1.17 (.87, 1.47)	1.29 (.95, 1.63)	8.6%	5.7%	**1.61 **(1.23, 1.99)^a^	**1.62 **(1.23, 2.02)^a^	25.0%	21.6%
Finance	**1.71 **(1.37, 2.06)^a^	1.37 (.91, 1.83)	28.6%	22.9%	1.22 (.91, 1.54)	1.35 (.99, 1.71)	5.6%	18.9%
Work/school	**1.60 **(1.18, 2.02)^a^	1.14 (.76, 1.53)	25.7%	14.3%	1.34 (.93, 1.76)	1.27 (.83, 1.71)	20.0%	29.7%
Family	**1.83 **(1.48, 2.18)^a^	1.29 (.94, 1.64)	31.4%	8.6%	**1.72 **(1.39, 2.05)^a^	**1.81 **(1.44, 2.17)^a^	25.0%	27.8%
Relationship separation	.37 (.12, .62)	.12 (−.03, .26)	2.9%	0%	.54 (.20, .89)	.38 (.11, .64)	5.7%	2.7%
Conflict with someone close	1.00 (.67, 1.33)	.51 (.25, .78)	3.1%	2.9%	1.19 (.81, 1.58)	**1.08 **(.71, 1.45)^a^	19.4%	10.8%
Important life events	.60 (.29, .91)	.46 (.12, .79)	5.7%	5.7%	.86 (.46, 1.26)	.92 (.53, 1.31)	13.9%	10.8%
Death or possible death of someone close	.51 (.20, .83)	.34 (.06, .63)	8.6%	5.7%	.78 (.39, 1.17)	1.05 (.63, 1.48)	8.3%	10.8%
Other stressors	1.00 (.53, 1.47)	.94 (.48, 1.40)	17.6%	17.1%	.81 (.42, 1.19)	.73 (.32, 1.14)	8.3%	13.5%

*Note*. Each source of stress was rated on a 5-point scale that included the following anchors: 0 (*none of the time*), 1 (*a little of the time*), 2 (*some of the time*), 3 (*most of the time*), and 4 (*all of the time*). Highly frequent stress was considered a rating of 3 or 4 (most or all of the time) on this rating scale.

^a^Means and confidence intervals that do not overlap with the corresponding mean rating and confidence intervals at that month in the persistently inactive symptom pattern group shown in Table 2 are indicated with this superscript and bold font.

## References

[1] Collins S. M. (2001). Stress and the gastrointestinal tract IV. Modulation of intestinal inflammation by stress: basic mechanisms and clinical relevance. *American Journal of Physiology—Gastrointestinal and Liver Physiology*.

[2] Bonaz B. L., Bernstein C. N. (2013). Brain-gut interactions in inflammatory bowel disease. *Gastroenterology*.

[3] Cohen S., Janicki-Deverts D. (2012). Who's stressed? Distributions of psychological stress in the United States in probability samples from 1983, 2006, and 2009. *Journal of Applied Social Psychology*.

[4] Bernstein C. N., Singh S., Graff L. A., Walker J. R., Miller N., Cheang M. (2010). A prospective population-based study of triggers of symptomatic flares in IBD. *The American Journal of Gastroenterology*.

[5] Keefer L., Keshavarzian A., Mutlu E. (2008). Reconsidering the methodology of ‘stress’ research in inflammatory bowel disease. *Journal of Crohn's and Colitis*.

[6] Singh S., Blanchard A., Walker J. R., Graff L. A., Miller N., Bernstein C. N. (2011). Common symptoms and stressors among individuals with inflammatory bowel diseases. *Clinical Gastroenterology and Hepatology*.

[7] Targownik L. E., Sexton K. A., Bernstein M. T. (2015). The relationship among perceived stress, symptoms, and inflammation in persons with inflammatory bowel disease. *American Journal of Gastroenterology*.

[8] Bernstein C. N., Blanchard J. F., Rawsthorne P., Wajda A. (1999). Epidemiology of Crohn's disease and ulcerative colitis in a central Canadian province: a population-based study. *American Journal of Epidemiology*.

[9] Longobardi T., Walker J. R., Graff L. A., Bernstein C. N. (2011). Health service utilization in IBD: comparison of self-report and administrative data. *BMC Health Services Research*.

[10] Clara I., Lix L. M., Walker J. R. (2009). The manitoba IBD index: evidence for a new and simple indicator of IBD activity. *American Journal of Gastroenterology*.

[11] Cohen S., Kamarck T., Mermelstein R. (1983). A global measure of perceived stress. *Journal of Health and Social Behavior*.

[12] Moradkhani A., Beckman L. J., Tabibian J. H. (2013). Health-related quality of life in inflammatory bowel disease: psychosocial, clinical, socioeconomic, and demographic predictors. *Journal of Crohn's and Colitis*.

[13] O'Leary C. J., Creamer D., Higgins E., Weinman J. (2004). Perceived stress, stress attributions and psychological distress in psoriasis. *Journal of Psychosomatic Research*.

[14] Gardner M. J., Altman D. G. (1986). Confidence intervals rather than P values: estimation rather than hypothesis testing. *British Medical Journal*.

[15] Cummings P., Koepsell T. D. (2010). P values vs estimates of association with confidence intervals. *Archives of Pediatrics and Adolescent Medicine*.

[16] Graff L. A., Walker J. R., Lix L. (2006). The relationship of inflammatory bowel disease type and activity to psychological functioning and quality of life. *Clinical Gastroenterology and Hepatology*.

[17] Lazarus R. S., Folkman S. (1984). *Stress, Appraisal, and Coping*.

[18] Becker H. M., Grigat D., Ghosh S. (2015). Living with inflammatory bowel disease: a Crohn's and Colitis Canada survey. *Canadian Journal of Gastroenterology and Hepatology*.

[19] Block J. P., He Y., Zaslavsky A. M., Ding L., Ayanian J. Z. (2009). Psychosocial stress and change in weight among US adults. *American Journal of Epidemiology*.

[20] Keller M. C., Neale M. C., Kendler K. S. (2007). Association of different adverse life events with distinct patterns of depressive symptoms. *The American Journal of Psychiatry*.

